# The efficacy and safety of Yupingfeng Powder with variation in the treatment of allergic rhinitis: Study protocol for a randomized, double-blind, placebo-controlled trial

**DOI:** 10.3389/fphar.2022.1058176

**Published:** 2022-12-16

**Authors:** Pui Kuan Cheong, Tin Muk Ho, Kam Leung Chan, Cho Wing Lo, Sin Bond Leung, Kam Lun Hon, Ka Chun Leung, Tony Hon Chung Siu, Tian-He Song, Hongwei Zhang, Jessica Yuet Ling Ching, Tak Yee Chow, Chi Him Sum, Chon Pin Chia, Zhi-Xiu Lin

**Affiliations:** ^1^ Hong Kong Institute of Integrative Medicine, The Chinese University of Hong Kong, Hong Kong, China; ^2^ S. H. Ho Centre for Digestive Health, Institute of Digestive Disease, Faculty of Medicine, The Chinese University of Hong Kong, Hong Kong, China; ^3^ School of Chinese Medicine, Faculty of Medicine, The Chinese University of Hong Kong, Hong Kong, China; ^4^ Department of Medicine and Geriatric, Alice Ho Miu Ling Nethersole Hospital, Hong Kong, China; ^5^ Department of Paediatrics, Faculty of Medicine, The Chinese University of Hong Kong, Hong Kong, China

**Keywords:** allergic rhinitis, Yupingfeng Powder, randomized control trial, Chinese medicine, IgE, efficacy and safety

## Abstract

**Background:** Allergic rhinitis (AR) is an upper airways chronic inflammatory disease mediated by IgE, which affects 10%–20% of the population. The mainstay for allergic rhinitis nowadays include steroids and antihistamines, but their effects are less than ideal. Many patients therefore seek Chinese medicine for treatment and Yupingfeng Powder is one of the most common formulae prescribed. In this study, we aim to investigate the efficacy and safety of Yupingfeng Powder with variation for the treatment of allergic rhinitis.

**Study design:** This is a double-blind, randomized, placebo-controlled trial. A 2-week screening period will be implemented, and then eligible subjects with allergic rhinitis will receive interventions of either “Yupingfeng Powder with variation” granules or placebo granules for 8 weeks, followed by post treatment visits at weeks 12 and 16. The change in the Total Nasal Symptom Score (TNSS) will be used as the primary outcome.

**Discussion:** This trail will evaluate the efficacy and safety of Yupingfeng Powder in treating allergic rhinitis. The study may provide the solid evidence of Yupingfeng Powder with variation can produce better clinical efficacy than the placebo granules.

**Trial registration:** ClinicalTrials.gov, identifier NCT04976023.

## 1 Introduction

Rhinitis, is a common disease generally defined as the inflammation of the nasal mucosa, affecting up to 40% of people (Small et al., 2007). The most common chronic rhinitis is allergic rhinitis (AR), which is affecting 10%–20% of people, and its prevalence is increasing globally (Dykewicz and Hamilos, 2010). AR can occur at any age and its occurrence is known to be related to patients’ living habits and environment (Wu and Mei, 2010; Li, 2015). Severe AR may affect quality of life, sleep and work performance of the patients (Dykewicz and Hamilos, 2010).

AR is an upper airways chronic inflammatory disease mediated by IgE, and the typical symptoms are sneezing, rhinorrhea, nasal itching, and nasal obstruction. AR is difficult to cure, and its relapse rate is also very high. If the condition is left untreated, it may give rise to various complications, such as sleep disturbances, general fatigue, loss of appetite, loss of attention and learning difficulty (Chen, 2013; Li, 2015).

The development of AR involves several factors, including the environment, immune system, and genetic susceptibility. Several types of cells, cytokines, and chemokines play important role in the allergic inflammation. Cytokines are responsible for mediating allergic inflammation, and the T helper cell (Th) 2 cytokines is essential in both the development of allergic sensitization and pathology of allergic inflammation (Maes et al., 2012). It has been shown that Th1-type cells are predominant in healthy subjects, while Th2-type cytokines are predominant in the nasal mucosa and epithelial tissues of AR subjects (Zhu and Paul, 2008). AR is a result of disequilibrium of Th1 and Th2 cytokines, for which Th2 is predominating over Th1 cells. Moreover, regulatory T-cell (Tregs), another subtype of T-cell, suppresses both the expression of Th1 and Th2-type cytokine (Akdis et al., 2006). And so, it has been suggested that there is an imbalance of Th2 and Treg-cell in AR patients (Sin and Togias, 2011). On the other hand, Tregs also restrict both Th1 and Th2 cell-mediated inflammation. The lack of Tregs may cause the happening of allergic inflammation with the increase of Th2 cytokines (Hawrylowicz and O’Garra, 2005). By synthesizing interleukin-10 (IL-10) and the transforming growth factor-β (TGF-β), Tregs can bring the allergic inflammation under control (Akdis et al., 2005). An allergen-specific functional defect of Treg is happening in AR patients, in whom the Tregs can promote the polarization of Th2, and consequently the synthesis of IgE (Han et al., 2010). IFN-γ, a characteristic cytokine produced by Th1 cells, is essential in bridging the innate and the adaptive arms of the immune system. In addition, IFN-γ is a key cytokine in the regulation of local leukocyte-endothelial interactions (Akkoc et al., 2008).

A previous study showed that a subset of CD4^+^ T cells, Th17 cells, is also important in the pathogenesis of allergic diseases (Fujiwara et al., 2007), which renders a new mechanism underlying the occurrence of AR. Th17 cell is responsible for the production of various cytokines, including IL-17, IL-6, TNF-α, and IL-22 (Schmidt-WeberCarsten et al., 2007; Wang and Liu, 2008). Studies suggests that during the acute phase of allergic reaction, neutrophilic infiltration may occur with Th17. IL-17 was found to involve in the induction of allergen-specific Th2 cell activation, eosinophil accumulation, and serum IgE production, thus suggesting IL-17A can regulate the established Th2-driven allergic immune response (Oboki et al., 2008). It has been found that serum IL-17 responses are correlated with the symptom severity scores, medication use, and peripheral eosinophil counts in AR patients (Ciprandi et al., 2008; Ciprandi et al., 2009). IL-10 is an important natural immune regulator and mainly produced by Th2 cells. It can inhibit the antigen-presenting effects of macrophages and the proliferation of active T cells, and suppress the intensity of inflammation (Wu et al., 2001; Levings et al., 2002).

In general, AR is a complicate immune and inflammatory disease. Its pathogenesis is not fully known yet, and there is no cure for this common allergic disorder (Zhou and Lu, 2010; Ni, 2011). Nowadays, the mainstay treatments for AR include steroids to reduce inflammation and swelling, and antihistamines to block the action of histamine (Ni, 2011). However, many clinical studies have found that the treatment effect of conventinal medicines is often undesirable, usually with adverse effects, such as nasal itching and frequent sneezing (Peng, 2014; Chen, 2015). Therefore, there exists a need to find a more effective and safer alternative for the treatment of AR.

Chinese herbal medicine (CHM) is a popular choice for AR patients seeking complementary and alternative therapies to reduce their clinical symptoms in East Asia (Chan and Chien, 2014; Kern and Bielory, 2014). Previous studies have shown that CHM could reduce nasal symptom, enhance quality of life, and strengthen body constitution in patients with AR (Luo et al., 2019). CHM appears to be a promising intervention for patients with AR. However, the previous studies had some methodological limitations, which weaken the clinical evidence for the efficacy of CHM. More scientifically methodological rigorous studies are needed to establish a solid evidence (Luo et al., 2019).

The core treatment principle of Chinese medicine are the syndrome differentiation and pattern identification. AR is in the disease category of “Rhinitis” in Chinese medicine. In the theory of Chinese medicine, this disease is resulted from the deficiency of Qi in lung, spleen and kidney, and is also due to the invasion of the external wind to the nasal orifices (Li, 2008; Zhou and Lu, 2010; Li, 2015). Therefore, according to tranditional Chinese Medicine (TCM) theory, the treatment principle of AR is to tonify the Qi of lung and spleen.

Yupingfeng Powder, composed of three Chinese herbs, i.e., *Astragalus mongholicus* Bunge [Fabaceae], *Atractylodes macrocephala* Koidz. [Asteraceae] and *Saposhnikovia divaricata* (Turcz. ex Ledeb.) Schischk. [Apiaceae], is commonly used for allergic diseases including AR (Yen et al., 2015). Clinical studies have shown that Yupingfeng Powder can improve symptoms of AR such as sneezing and nasal itching (Chan and Chien, 2014; Fang et al., 2014) and quality of life (Chan and Chien, 2014) and decrease the levels of interleukins like IL-4 and IgE in AR patients (Fang et al., 2014). A glucosidic extract of Yupingfeng Powder was found to have the ability to activate the T helper cells and regulate some subsets of T lymphocytes, which can exert anti-inflammatory and immunoregulatory (Gao et al., 2009).

Oral CHM influences the gastrointestinal system, including the gut microbiota and the intestinal mucosa. The gut microbiota has been found highly related to our health and various diseases (Frick and IngoAutenrieth, 2012). Studies show that certain probiotics can improve the symptoms of AR patients (Harissios et al., 2008; Nagata et al., 2010; Costa et al., 2014; Güvenç et al., 2016). Thus, we hypothesize that gut microbiota may be influenced by complex interplays with the CHM components, such as glycosides and oligosaccharides (Xu et al., 2017), in the same manner as an oral probiotic to achieve systematic immunological conditions by regulating local intestinal immunological conditions as shown in previous studies (Ivory et al., 2008; Levy et al., 2017).

In recent years, some clinical studies have found that Yupingfeng Powder can increase anti-allergic ability and improve body’s resistance with few side effects, such as feeling of abdominal distension or increase in acne (Chen, 2013; Chan and Chien, 2014), indicating the advantages of oral Chinese medicine in the treatment of AR. In this project, we designed a double-blind, randomized, placebo controlled clinical trial to assess the efficacy of Yupingfeng Powder with variation for AR patients with TCM syndrome of qi deficiency of lung and spleen, and to explore the molecular mechanism of Yupingfeng Powder in treating AR with respect to the modulation of immune response and gut microbiota.

## 2 Methods

### 2.1 Study design

This is a randomized, double-blind, placebo-controlled trial. A 2-week screening period will be implemented, and then eligible subjects with AR will receive either “Yupingfeng Powder with variation” granules or placebo granules for 8 weeks followed by a post treatment visits at weeks 12 and 16.

### 2.2 Study population

Potentially eligible subjects with AR will be screened for the following eligibility criteria:

#### 2.2.1 Inclusion criteria

• Aged 5 or above;
• Subjects with deficiency of lung and spleen Qi, which will be determined by Chinese Medicine Practitioners (CMP). (There will be a checklist of 13 TCM symptoms, subjects with two or more of the symptoms will be determined as deficiency of lung and spleen Qi);
• At least two or more allergic symptoms (rhinorrhea, sneezing, nasal obstruction and nasal itching) for a cumulative period greater than 1 h per day. These symptoms may be accompanied by itchy and red eyes and tears; and
• Able to provide voluntary written informed consent.

#### 2.2.2 Exclusion criteria

• Known chronic disease such as asthma, rhinosinusitis, nasal polyposis;
• Known severe medical conditions, such as cardiovascular, liver or renal dysfunction, diabetes mellitus, cancers, cerebrovascular diseases, blood system diseases;
• Concomitant use of steroid, non-steroidal anti-inflammatory drugs (NSAIDs), anticoagulant, and immunotherapy within past month;
• Impaired hematological profile and liver/renal function exceeds the upper limit of the reference value by two times;
• Known alcohol and/or drug abuse;
• Known allergic history to any Chinese herbal medicines; or
• Known pregnant or lactating.

### 2.3 Study intervention

#### 2.3.1 Study treatments

The eligible subjects will be randomly allocated to receive either “Yupingfeng Powder with variation” granules (15.55 g twice daily) or placebo granules (15.55 g twice daily) for 8 weeks. Variable dosages will be adopted according to the age of subjects. The daily dosage of “Yupingfeng Powder with variation” consists of the following herbal formula: *A. mongholicus* Bunge [Fabaceae] (Huang-Qi) 20 g, *A. macrocephala* Koidz. [Asteraceae] (Bai-Zhu) 10 g, *S. divaricata* (Turcz. ex Ledeb.) Schischk. [Apiaceae] (Fang-Feng) 6 g, *Magnolia biondii* Pamp. [Magnoliaceae] (Xin-Yi-Hua) 10 g, processed *Xanthium strumarium* L. [Asteraceae] (Chao-Cang-Er-Zi) 8 g, *Ganoderma lucidum* (Chi-Ling-Zhi) 12 g, *Platycodon grandiflorus* (Jacq.) A.DC. [Campanulaceae] (Ji-Geng) 10 g, processed *Drgonsbones* (Duan-Long-Gu) 15 g, processed *Ostreae concha* (Duan-Mu-Li) 15 g, *Terminalia chebula* Retz. [Combretaceae] (He-Zi) 8 g, *Glehnia littoralis* (A.Gray) F.Schmidt ex Miq. [Apiaceae] (Bei-Sha-Shen) 10 g, *Cordyceps sinesis* powder (Chong-Cao-Jun-Si-Fen) 3 g, *Dictamnus dasycarpus* Turcz. [Rutaceae] (Bai-Xian-Pi) 10 g. The study drug will be prepared using single granules, with the ratio of 1:3 for *A. macrocephala* Koidz. [Asteraceae] and 1:5 for the others according to the granules preparation method of the Nong’s Company Limited, i.e., 1 g granule = 3 g decoction piece and 1 g granule = 5 g decoction piece respectively, except for *Cordyceps sinesis* powder which is in the ratio of 1:1. The above granules are all extraction by water of the crude plants.

The granules are commercially available. The quality control and production of Nong’s Company Limited comply with the GMP standards of the PRC, Australia and the USP. All the herbal medicines comply with the standards of Chinese Pharmacopoeia. There is also laboratory for quality control with ISO 17025 certificate.

One daily dose of “Yupingfeng Powder with variation” granules will be equally sealed into two packs, which will be dissolved in hot water and administered while it is lukewarm. Participants will take two times daily, one pack in the morning and another in the evening after meal. Those subjects aged 5–12 will take half of the total dose, and those aged 13 or above will take normal adult dose.

The placebo granules will be prepared from starch and an edible pigment and will be matched as closely as possible to the appearance and taste of “Yupingfeng Powder with variation” granules.

All study medications (“Yupingfeng Powder with variation” granules and placebo granules) will be produced by the manufacturer following good manufacturing practice. They will be packed at the Pharmacy of the Integrative Medical Center of the Hong Kong Institute of Integrative Medicine, The Chinese University of Hong Kong.

#### 2.3.2 Prohibited drugs

Anticoagulant agents, NSAIDs, steroids, antihistamine, immunotherapy, antibiotics, prebiotics, probiotics and Chinese herbal products which are used to treat AR are prohibited during the study period.

#### 2.3.3 Treatment compliance

Drug compliance is assessed by the number of doses taken during study period by subjects’ self-report.

### 2.4 Patient visits

#### 2.4.1 Screening visit

Information about the study, processing and scheduling will be explained before enrolment. Subjects will be screened within 14 days prior to treatment commencement. Medical consultation and assessment, medical history, vital sign and concomitant medication will be assessed and recorded. Blood samples for serum IgE, complete blood picture (CBP) with differentiation and renal and liver function will also be taken at screening visit. The screening assessment can be served as the baseline assessment. Informed consent will also be obtained after the study has been fully explained to each subject.

#### 2.4.2 Baseline randomization visit

Eligible subjects will have medical consultation and assessment by Registered Chinese medicine practitioner (CMP) investigators with at least 3 years’ clinical experience. Vital sign will be assessed, medical history and concomitant medications will also be recorded at baseline. TNSS, Rhinoconjunctivitis Quality of Life Questionnaire (RQLQ) score for aged 13 and above and Paediatric Allergic Disease Quality of Life Questionnaire (PADQLQ) score for aged 12 and Visual Analog Scale (VAS) for frequency and severity of AR episodes will be evaluated. Blood samples for serum cytokines (IL-10 and IL-17) will also be collected. Subjects will be randomly assigned (in a 1:1 ratio) to receive for 8 weeks of either “Yupingfeng Powder with variation” granules or placebo granules.

#### 2.4.3 Follow-up visit

CMP investigators with at least 3 years’ clinical experience will provide consultation under Chinese medicine theory, prescribe and dispense “Yupingfeng Powder with variation” granules or placebo in this trial. Subjects will return for follow-up at baseline, weeks 4 (±4 days) and 8 (±4 days), and followed by a post-treatment visit at weeks 12 and 16 (±4 days) (Table 1).

**TABLE 1:** Study schedule.

Items	Screening	Treatment period			Post-treatment follow up	
	−14 to 0 days	Day 0 (baseline)	Week 4	Week 8	Week 12	Week 16
			(±4 days)	(±4 days)	(±4 days)	(±4 days)
Clinical assessment						
Informed consent	X					
Eligibility	X					
Medical consultation and assessment	X	X	X	X	X	X
Medical history	X					
Concomitant medication	X	X	X	X	X	X
Vital sign (BP/pulse)	X	X	X	X	X	X
AE/SAE assessment			X	X	X	X
Outcome assessment						
Total nasal symptom score (TNSS)		X	X	X	X	X
Visual analog scale (VAS)		X	X	X	X	X
Rhinoconjunctivitis Quality of Life Questionnaire (RQLQ) and Paediatric Allergic Disease Quality of Life Questionnaire (PADQLQ)		X	X	X	X	X
Serum level of IgE	X			X	X	
Serum levels of Cytokines (IL-10 and IL-17)		X		X	X	
Gut microbiota		X		X		
Laboratory assessment						
CBP with differentiation	X			X		
Renal and liver function test	X			X		
Intervention						
Administration of study drug		X	X	X		

### 2.5 Randomization and blinding

The eligible subjects will be randomly assigned to either experimental group or control group at the ratio of 1:1. A randomization schedule will be generated by computer to assign subjects to the treatment sequences. Concealment of allocation will be ascertained by an independent research staff, and identically designed treatment packs will be used for study drugs.

This is a double-blind randomized controlled trial. All study investigators and subjects will be blinded in the study. The random allocations will be put into opaque envelops with sequential study numbers. Two sets of the envelope will be prepared, with one set for randomization at the site, and another set for storage in the investigator’s office for emergency un-blinding.

### 2.6 Assessments

The changes of TNSS, RQLQ score for aged 13 and above and PADQLQ score for aged 5–12 and frequency and severity of AR episodes (VAS) will be evaluated at each study visit. Also vital sign and concomitant medications will be assessed and recorded. Subjects’ self-reported Chinese medicine adherence will be assessed, and reasons for skipping doses will be recorded.

Blood samples for serum cytokines (IL-10 and IL-17) will be collected at baseline, weeks 8 (±4 days) and 12 (±4 days). Stool specimen (within 24 h of the study visit day) will be collected at baseline and week 8 (±4 days). Subjects’ information will be kept anonymous.

Assessment of safety will be conducted by laboratory parameters and subjects’ reported adverse events and/or serious adverse events. The assessment of adverse event will be recorded according to CTCAE 4.0 which is a standard assessment tool. A direct telephone line will be provided so that subjects can report any adverse events during office hours between scheduled visits. The subjects will be recommended to attend Emergency Department at the nearest hospital beyond office hours if deemed necessary.

All subjects will be required to keep daily record for monitoring their compliance and side effects of the study treatment. Besides, they need to record 3 days of food diary before the day for taking stool specimens for gut microbiome analysis as the food intake may affect the gut species and influence the gut microbiome analysis result.

### 2.7 Subject withdrawal

A subject must be withdrawn from the study if he/she withdraws consent. Subjects who 1) experience adverse events, or 2) have pre-existing violation of entry criteria may remain in the study unless the investigator determines that it is not in the subject’s best interest to continue. The specific reason for withdrawal should be indicated.

Subjects who have withdrawn from the study will be invited to follow up at the last study visit, i.e., week 16 after the randomization visit to detect any delayed clinical events.

## 3 Study outcomes

### 3.1 Primary outcomes

1. The change in the TNSS at week 8.

### 3.2 Secondary outcomes

1. The change in the TNSS at weeks 4, 12, and 16.
2. The changes in frequency of AR episodes and their severity VAS at weeks 4, 8, 12, and 16;
3. The changes in the RQLQ score or PADQLQ score;
4. The changes in the serum levels of total IgE and cytokines (IL-10 and IL-17) at weeks 8 and 12;
5. The changes in the gut microbiota composition in stools at week 8; and
6. Adverse events (graded by CTCAE) related to study treatment.

### 3.3 Study assessment

#### 3.3.1 Nasal symptom score

The TNSS will be self-assessed by subjects to evaluate four nasal symptoms (rhinorrhea, sneezing, nasal obstruction, and nasal itching), using a four-point scale as shown (Pfaar et al., 2014):

0=no symptoms

$$1=mild\;symptoms\;\left(sign/symptom\;clearly\;present,\;but\;minimal\;awareness;\;easily\;tolerated\right)$$

$$2=moderate\;symptoms\;\left(definite\;awareness\;of\;sign/symptom\;that\;is\;bothersome\;but\;tolerable\right)$$

$$3=severe\;symptoms\;\left(sign/symptom\;that\;is\;hard\;to\;tolerate;\;causes\;interference\;with\;activities\;of\;daily\;living\;and/or\;sleeping\right)$$

The higher the score, the more severity of the symptom.

#### 3.3.2 Visual analog scale (VAS)

The VAS ranges from 0 (nasal symptom free) to 10 (nasal symptom extremely bothersome) to assess the severity of nasal symptom disturbance and has been validated for use in the quantitative evaluation of AR severity (Bousquet et al., 2007). The higher the score, the more severity of the symptoms.

#### 3.3.3 Rhinoconjunctivitis quality of life questionnaire (RQLQ) for aged 13 and above

The RQLQ is a disease-specific questionnaire consists of 28 questions covering seven domains including activities, sleep, non-nose/eye symptoms, practical problems, nasal symptoms, eye symptoms and emotional problems, that can assess quality of life impairment in AR patients. This questionnaire (Juniper and H Guyatt, 1991).

#### 3.3.4 Paediatric allergic disease quality of life questionnaire (PADQLQ) for aged 5–12

The PADQLQ is a questionnaire designed for children answering without the parental help. It consists of 26 questions with three domains: activities (eight questions), symptoms (15 questions), and emotions (three questions). For each question, the subjects are asked for the perceived bother due to their allergic diseases. A 6-point scale from “not bothered” (score = 0) to “extremely bothered” (score = 6) is used to recording the perceived degree of bother. The total score of the PADQLQ is derived by summation of the scores for all 26 questions. The score for each of the domains is also calculated by summation of the relevant scores (Ng et al., 2011).

#### 3.3.5 Serum levels of cytokines and IgE

Blood samples will be taken to detect the serum level of cytokines IL-10 and IL-17 and specific IgE using enzyme-linked immunosorbent assays (ELISAs). These are the markers for inflammatory response. The higher the value, the more severity of the symptoms.

#### 3.3.6 Composition of gut microbiota

Stool samples will be collected to detect bacterial taxa present by using ribosomal ribonucleic acid (rRNA) sequencing to observe the influence of Chinese medicines on the composition of the gut microbiota. This experiment is exploratory in nature and no specific microbiota is known.

## 4 Possible risk and adverse event reporting

### 4.1 Possible risks and discomfort

There is minimal discomfort in blood taking. No serious adverse event has ever been reported but only mild discomfort of taking Yupingfeng Powder, including feeling of abdominal distension or increase in acne (Chan and Chien, 2014). The adverse reaction will be recorded and the treatment will be suspended when severe adverse reaction occurs. Un-blinding may also be done if necessary when severe adverse events occur.

### 4.2 Adverse events

An adverse event is any undesirable medical event occurring in the subject within the trial period, whether or not it is related to the study intervention. The assessment of adverse event will be recorded according to CTCAE 4.0 which is a standard assessment tool.

### 4.3 Serious adverse events

A serious adverse event is an adverse event that results in one of the following outcomes:

• Death
• Life-threatening
• In-patient hospitalization or prolongation of existing hospitalization
• A persistent or significant disability or incapacity
• A congenital anomaly or birth defect

The definitions of causal relationship to study intervention are the same as those for adverse events. A standard serious adverse event form will be used (provided by The Chinese University of Hong Kong—New Territories East Cluster Combined Research Ethics Committee [Joint CUHK-NTEC CREC] at http://intranet.ccter.cuhk.edu.hk/sae/) to report the events within 24 h after acknowledgement.

## 5 Sample size and statistical methods

### 5.1 Sample size calculation

This study is mainly focused on determining the efficacy of “Yupingfeng Powder with variation” for AR patients. The primary outcome is the TNSS. With reference to data from a previous similar study (Chen, 2015), the sample size was calculated using two means with software G*Power. The mean of nasal score of the treatment group was 0.5 ± 0.1 and that of the control group was 3.6 ± 0.2. To obtain a significance level of 5%, a power of 80%, 52 subjects are required. Allowing for a 10% dropout rate, 58 subjects are needed, with 29 in each group.

### 5.2 Statistical analysis

Baseline data (gender, age, vital signs) will be descriptively summarized. Differences of measurement data between the groups will be assessed using *t*-test for normally distributed continuous variables and Wilcoxon signed rank test for non-normally distributed variables.

Measurement data of different groups in each visit will be reported as mean ± standard deviation (SD). Intra-group comparisons between baseline and each visit will be conducted by using paired *t*-test (or Wilcoxon signed rank test). Comparisons between groups will be conducted by using an analysis of variance (ANOVA), with other confounding factors like multicenter character conducting the covariate analysis. Statistical analysis for the data which do not meet above conditions (e.g., non-normal) will be conducted with the use of non-parametric test.

For AEs analysis, comparisons of the incidence rate of AEs between groups will be conducted with the use of χ^2^ test. The investigators need to list and describe the AEs occurred in this trial. If the data do not conform to χ^2^ test (data include 0, or theoretical frequency is below 5), Fisher’s exact test will be used.

For laboratory parameters, Spearman’s coefficient correlation will be used for comparison with different parameters and one way ANOVA test for significant correlation between groups.

All *p* values and 95% confidence intervals are two-sided, and *p* < 0.05 is considered statistically significant. Statistical analyses will be carried out exclusively by an independent statistician. All analyses will be performed by using SPSS, version 24 (SPSS).

## 6 Ethics consideration

This study complies with the Declaration of Helsinki in ICH-GCP. Application for ethical approval will be sought to The Chinese University of Hong Kong before the initiation of this study. Participants and their parents will be informed of ensuring the confidentiality of all information and data will be maintained anonymity. Consent forms should be signed by the subjects’ parents (under 18 years of age) or subjects (over 18 years of age) before the study. All information will be encrypted and only the involved investigators can access. Password is required to access the data. Participants are free to withdraw at any time without giving a reason or punishment. The personal data of the subjects will only be kept for 10 years and will be destroyed afterwards.

## 7 Quality assurance

### 7.1 Data management

Only the study team and the PI have access right to the database using their login user names and passwords. Subjects’ information is kept anonymous. They are only identified by their study numbers to maintain subjects’ confidentiality.

### 7.2 Auditing and inspection

The Clinical Research Management Office at The Chinese University of Hong Kong or an external auditor will perform auditing or inspection to determine whether research activities are conducted according to the protocol, Good Clinical Practice (GCP) and guidelines of the International Conference on Harmonization (ICH).

## 8 Discussion

AR is a common disease (Dykewicz and Hamilos, 2010) that may affect the quality of life, sleep and work performance of the patients (Dykewicz and Hamilos, 2010). However, current available treatments for AR mainly include steroids and antihistamine (Ni, 2011), and the therapeutic effects are not always desirable (Peng, 2014; Chen, 2015). For this reason, many AR patients often seek help from Chinese herbal medicine, which has been shown to be effective in managing the patients’ symptoms. Chinese herbal medicine poses to be a valuable alternative for AR (Luo et al., 2019).

Yupingfeng Powder is a well-known formula in TCM with a long history, and is commonly used to treat allergic disease (Chan and Chien, 2014; Yen et al., 2015). The results from a few clinical trials showed that Yupingfeng Powder on top of conventional medicine brought about high efficacy than conventional medicine alone in treating AR (Zhou and Lu, 2010; Ni, 2011; Peng, 2014; Chen, 2015). However, the clinical study on the efficacy and safety of Yupingfeng Powder alone in treating AR is few and far between. The proposed project aims to determine whether Yupingfeng Powder is effective and safe in managing AR.

It should be noted that in this trial we have modified the original formula of Yupingfeng Powder. The original formula was designed to improve the immunity and tonifying the Lung and Spleen Qi. In this study, several additional herbal medicines are added to the formula to strengthen the functions of the original formula. For example, *M. biondii* (Xin-Yi-Hua), processed *X. strumarium* (Chao-Cang-Er-Zi) and *P. grandiflorus* (Ji-Geng) are added to relieve the nasal congestion of AR, while Processed Dragons bones (Duan-Long-Gu), processed *O. concha* (Duan-Mu-Li), *D. dasycarpus* (Bai-Xian-Pi) and *T. chebula* (He-Zi) have the ability to reduce sneeze and nasal discharge often seen in AR patients. *G. lucidum* (Chi-Ling-Zhi) and Cordyceps sinesis powder (Chong-Cao-Jun-Si-Fen) are used to increase the overall immunity of the body, while *G. littoralis* (Bei-Sha-Shen) enhances the Lung qi tonifying effect of the original formula. The modified formula is specifically designed for AR, and therefore is expected to produce better efficacy on the treatment of AR.

It has been known that there is an imbalance of Th1-type and Th2- type cytokines, with Th2-type cytokines dominated over Th1-type cytokines (Zhu and Paul, 2008). And Tregs also play an important role by synthesizing IL-10 to suppress the expression of both Th1 and Th2-type cytokines (Akdis et al., 2006). IL-17, which is produced by Th17 cells, was found to contribute to the induction of the activation of Th2 (Oboki et al., 2008). Previous study already shows that IL-10 and IL-17 were detected higher in AR subjects’ serum than healthy individuals (Bayrak Degirmenci et al., 2018). And therefore we will observe the change in serum level of cytokines IL-10 and IL-17. Also IgE, and the gut microbiota composition will be used as outcome measures, and these clinical outcomes are relevant to clinical reality of AR management. We are confident that through this trial, we would be able to establish much-needed clinical evidence about the efficacy and safety of Yupingfeng Powder with variation in the treatment of AR.

## 9 Trial status

The participants are currently being recruited for the present study. We have recruited 11 participants currently on 18 July 2022.

## Abbreviations

AR, allergic rhinitis; TNSS, Total Nasal Symptom Score; Th, T helper cell; Tregs, regulatory T-cell; IL, interleukin; TGF- β, transforming growth factor- β; CHM, Chinese herbal medicine; TCM, Traditional Chinese Medicine; CMP, Chinese Medicine Practitioner; NSAIDs, |non-steroidal anti-inflammatory drugs; CBP, complete blood picture; RQLQ, Rhinoconjunctivitis Quality of Life Questionnaire; PADQLQ, Paediatric Allergic Disease Quality of Life Questionnaire; VAS, Visual Analog Scale; ELISAs, enzyme-linked immunosorbent assays; rRNA, ribosomal ribonucleic acid; SD, standard deviation; ANOVA, analysis of variance; GCP, Good Clinical Practice; ICH, International Conference on Harmonization.
